# Editorial: Limbic-Brainstem Roles in Perception, Cognition, Emotion, and Behavior

**DOI:** 10.3389/fnins.2018.00395

**Published:** 2018-06-12

**Authors:** Hisao Nishijo, Robert Rafal, Marco Tamietto

**Affiliations:** ^1^System Emotional Science, Graduate School of Medicine, University of Toyama, Toyama, Japan; ^2^Department of Psychological and Brain Sciences, University of Delaware, Newark, DE, United States; ^3^Department of Medical and Clinical Psychology, Center of Research on Psychology in Somatic Diseases, Tilburg University, Tilburg, Netherlands; ^4^Department of Psychology, University of Torino, Torino, Italy; ^5^Netherlands Institute for Advanced Study in Humanities and Social Sciences, Royal Netherlands Academy of Arts and Sciences (KNAW), Amsterdam, Netherlands

**Keywords:** brainstem, subcortical pathway, limbic system, emotion, behavior, cognition, memory

Converging and extensive evidence suggests that the limbic-brainstem regions receive direct perceptual information bypassing early sensory cortical systems and play a central role in innate behaviors, including motivated and avoidance behaviors. Recent studies in human patients with cortical blindness as well as in healthy participants suggest that these subcortical sensory pathways are functional in the intact human brain and interact with more evolutionary recent cortical systems (Celeghin et al., 2017). Phylogenetic continuity also indicates that such subcortical systems present in human and non-human primates might be present in other species, whereby they underlie similar functions. For example, birds seem to have similar subcortical neural circuits to those involved in facial recognition in humans. These studies provided substantial evidence for a rapid, coarse, subcortical sensory systems including the superior colliculus, pulvinar, and amygdala (Le et al., 2018).

Furthermore, the brain is composed by several hierarchical networks from the higher cortical systems to the lower brainstem, and these systems are intimately interconnected (Dinh et al., 2017). Therefore, brainstem lesions could affect higher functions in the cortical areas and breakdown object recognition, attention, personality, learning and memory, social cognition, consciousness, etc. Taken together, these findings suggest that dysfunction in the limbic-brainstem regions is associated with various psychiatric disorders with higher cognitive deficits including autism, schizophrenia, face blindness (prosopagnosia), attention deficits, hyperactivity disorder (ADHD), phobia, etc.

The aim of this research topic is to present up-to-date advancements in this area and to highlight the functions of the limbic-brainstem regions in a variety of perceptual, cognitive, affective, and behavioral domains. The special issue offers an inter-disciplinary approach to these topics, including cross-species studies in human and non-human primates. Chapter 1 focuses on visual processing in the subcortical areas. Diano et al. provide a comprehensive review of amygdala role in unconscious visual processing of emotional stimuli and the multiple neural pathways involved. Bourne and Morrone provide a review on the role of the pulvinar in promoting rapid development as well as functional plasticity in the cortical visual system after lesions to the geniculostriate pathway in early life. Bollini et al. investigated event-related potentials (ERPs) in a patient with a lesion involving V1 and blindsight. Interestingly, they found early as well as late ERP components that likely reflect the electrophysiological correlates of different aspects of unconscious visual processing. van Koningsbruggen et al. investigated redundant target effects (RTE), in which two visual targets presented simultaneously are more rapidly detected than a single target. They reported that two patients with lesions in the brachium of the SC showed no RTE effect. This is in line with previous reports (Savazzi and Marzi, 2004; Tamietto et al., 2010) and provides direct evidence of a causal contribution of the SC to the RTE. Framorando et al. investigated VEPs in response to task-irrelevant fearful faces. They reported that fearful faces presented in the temporal visual field, but not nasal visual field, induced distractor effects in the VEPs. This finding suggests that a putative subcortical pathway from the superior colliculus to the amygdala, via the pulvinar, which signals fearful faces, receives direct input to the colliculus from the retina via the retino-tectal tract. Yoshida et al. reported that the V1-lesioned monkeys could use pre-cues (arrows) predicting location of upcoming targets in the affected hemifield, consistent with human blindsight patients who can direct attention in cueing paradigms. Nguyen et al. investigated monkey SC and pulvinar neuronal responses to human facial photos, and reported that ensemble activity of SC and pulvinar neurons discriminated gender, head orientation, and identity.

Chapter 2 focuses on the role of the subcortical areas including the thalamus and brainstem in behavioral manifestation. Soares et al. provide a critical review on the role of the subcortical visual pathway in forming responses to emotionally-charged biologically-relevant stimuli (snakes, fearful, and aggressive faces) and in visually guided reaching and grasping in primates. They proposed that the subcortical pathway might have evolved as a rapid detector/first responder. Ichijo et al. review the role of the habenula (Hb) in functional lateralization of binary opposite behavioral manifestation (e.g., escaping/freezing, winning/loosing) in vertebrates. They proposed that the lateralized asymmetrical circuits might have evolved under natural selection pressure based on functional incompatibility of binary opposite behaviors (e.g., the neural system for escaping is not useful to freezing). Forcelli et al. investigated a role of the periaqueductal gray (PAG) of monkeys in defensive behaviors by chemical stimulation. They reported that PAG stimulation induced defense-associated vocalization and postural/locomotor asymmetry, but not motor defense responses, suggesting functional dissociation between the PAG and SC, stimulation of which induces motor defense responses. Okada and Kobayashi investigated tonic neuronal responses in the pedunclopontine tegmental nucleus (PPTg) of monkeys in a visually guided saccade task. They found two types of neurons with tonic responses; responses associated with reward prediction and those associated with attention to target stimuli, suggesting a PPTg involvement in not only reinforcement learning but also execution of conditioned behaviors. Terao et al. investigated effects of saccade intrusions on subsequent oculomotor and motor responses in healthy subjects and patients with Parkinson's disease (PD). They reported that saccade intrusions delayed subsequent responses in normal subjects while opposite results were observed in the PD subjects with long latencies beyond the normal range, and discuss its neural mechanisms involving complex cortical system-basal ganglia-SC circuits. Puviani et al. review and propose that pupillometry can be used to evaluate activity of subcortical limbic-brainstem circuits, which might be associated with various psychiatric diseases such as schizophrenia, phobia, PTSD, ADHD, addiction, etc.

Chapter 3 handles integrated roles of the limbic system and thalamus. Van Mao et al. provide a comprehensive review of the role of the pregenual part of the anterior cingulate gyrus of primates in social cognition and spontaneous social interaction. Duquette reviews neural mechanisms of “interoception,” feeling based on body homeostasis, and suggest that interoceptive disturbance might be associated with various psychiatric diseases such as depression, anxiety, etc., and psychotherapy such as mindfulness could ameliorate interoceptive disturbance. Chen et al. investigated human amygdala using fMRI, and found that subjects who used expressive suppression more frequently showed less amygdalar activation in response to negative conditioned stimuli. Matsumoto et al. investigated neuronal responses to ultrasonic vocalization (USV) in the rat amygdala, and reported that amygdala neurons responded preferentially to USV emitted by other rats rather than own USV. They suggest that hyperactivation of these neurons might induce auditory hallucination of other' voice. Harvey et al. reported that post-training inactivation of the anterior thalamic nuclei (ATN) decreased spatial discrimination in a radial-arm maze in rats, suggesting that the ATN is required for expression of previously acquired spatial representation in radial environments. Sun et al. investigated effects of up- and down-regulation of miR-30c, which is involved in promotion of adult neurogenesis, in the subventricular zone (SVZ) and dentate gyrus (DG), and reported significant morphological and functional changes associated with the olfactory bulb and hippocampus, suggesting that certain amount of newborn neurons are required for morphological and functional maintenance of these regions.

In conclusion, the subcortical limbic-brainstem might function as a hub to form shortcut sensory (especially visual)-motor (or autonomic) circuits that allow rapid innate behaviors. The subcortical visual pathway in this circuit might also support various phenomena in blindsight patients with V1 lesions (Tamietto and Morrone, 2016) unconscious emotional processing (Burra et al., 2018), stimulus detection by redundant target effects (RTE), unconscious cuing effects, some forms of stimulus discrimination, etc. Furthermore, the limbic-brainstem is involved in integrated functions such as social interaction, interoception, emotion regulation, spatial discrimination, etc., which might be manifested under an interaction between the subcortical and cortical systems. We hope that this current research topic provides a comprehensive review to understand roles of the subcortical limbic-brainstem in some forms of sensory-motor coupling, cognitive and affective functions.

## Author contributions

All authors listed have made a substantial, direct and intellectual contribution to the work, and approved it for publication.

### Conflict of interest statement

The authors declare that the research was conducted in the absence of any commercial or financial relationships that could be construed as a potential conflict of interest.

## References

[B1] Burra N. Hervais-Adelman A. Celeghin A. de Gelder B. Pegna A. J. (2018). Affective blindsight relies on low spatial frequencies. Neuropsychologia. [Epub ahead of print]. 10.1016/j.neuropsychologia.2017.10.009 28993236

[B2] Celeghin A. Diano M. de Gelder B. Weiskrantz L. Marzi C. A. Tamietto M. (2017). Intact hemisphere and corpus callosum compensate for visuomotor functions after early visual cortex damage. Proc. Natl. Acad. Sci. U.S.A. 114, E10475–E10483. 10.1073/pnas.1714801114 29133428 PMC5715784

[B3] Dinh H. T. Nishimaru H. Matsumoto J. Takamura Y. Le Q. V. Hori E. (2017). Superior neuronal detection of snakes and conspecific faces in the macaque medial prefrontal cortex. Cereb. Cortex 28, 2131–2145. 10.1093/cercor/bhx118 28498964

[B4] Le Q. V. Nishimaru H. Matsumoto J. Takamura Y. Nguyen M. N. Mao C. V. (2018). Gamma oscillations in the superior colliculus and pulvinar in response to faces support discrimination performance in monkeys. Neuropsychologia. [Epub ahead of print]. 10.1016/j.neuropsychologia.2017.10.015 29037507

[B5] Savazzi S. Marzi C. A. (2004). The superior colliculus subserves interhemispheric neural summation in both normals and patients with a total section or agenesis of the corpus callosum. Neuropsychologia 42, 1608–1618. 10.1016/j.neuropsychologia.2004.04.011 15327929

[B6] Tamietto M. Cauda F. Corazzini L. L. Savazzi S. Marzi C. A. Goebel R. (2010). Collicular vision guides nonconscious behavior. J. Cogn. Neurosci. 22, 888–902. 10.1162/jocn.2009.21225 19320547

[B7] Tamietto M. Morrone M. C. (2016). Visual plasticity: blindsight bridges anatomy and function in the visual system. Curr. Biol. 26, R70–R73. 10.1016/j.cub.2015.11.026 26811892 PMC5172419

